# Benefits of Patisiran on Functional Capacity in ATTR Cardiac Amyloidosis: Post Hoc Analysis of APOLLO-B

**DOI:** 10.1016/j.jacadv.2025.101876

**Published:** 2025-06-23

**Authors:** John L. Berk, Olivier Lairez, Pedro Schwartzmann, Shaun Bender, Matthew T. White, Patrick Y. Jay, David Danese, Ronald Witteles

**Affiliations:** a Boston University School of Medicine, Boston, Massachusetts, USA; b Hôpital de Rangueil, Toulouse, France; c Advanced Research Centre (CAPED) and Unimed Hospital, Ribeirão Preto, São Paulo, Brazil; d Alnylam Pharmaceuticals, Cambridge, Massachusetts, USA; e Stanford University School of Medicine, Stanford, California, USA

**Keywords:** ATTR amyloidosis, cardiomyopathy, functional capacity, heart failure, RNAi therapeutics

## Abstract

**Background:**

Patients with transthyretin amyloidosis with cardiomyopathy (ATTR-CM) suffer substantial morbidity and mortality. The meaningful preservation of functional capacity and quality of life are important priorities.

**Objectives:**

The 6-minute walk test (6MWT), a measure of functional capacity, was the primary outcome in the APOLLO-B study of patisiran in patients with ATTR-CM; the treatment benefit vs placebo was +15 m over 12 months. We estimated the minimal clinically important difference for change in 6MWT performance.

**Methods:**

Change from baseline in 6MWT performance was anchored to established categories of clinically important change in the Kansas City Cardiomyopathy Questionnaire-Overall Summary score. To relate changes in 6MWT performance to activities of daily living, we fit a proportional-odds cumulative logit model for items in the Kansas City Cardiomyopathy Questionnaire Physical Limitation domain.

**Results:**

The APOLLO-B trial randomized 360 patients to receive placebo (n = 179) or patisiran (n = 181). The estimated minimal clinically important difference in 6MWT was 6.9 to 7.8 m. When comparing the change from baseline in 6MWT at month 12 between 2 patients, 15 m greater preservation was associated with approximately 10% to 16% lower odds of deterioration in walking 1 block (OR: 0.88 [95% CI: 0.83-0.93]), climbing stairs (OR: 0.84 [95% CI: 0.80-0.89]), hurrying/jogging (OR: 0.88 [95% CI: 0.83-0.93]), dressing oneself (OR: 0.85 [95% CI: 0.81-0.90]), and performing yard/housework or carrying groceries (OR: 0.89 [95% CI: 0.84-0.93]).

**Conclusions:**

In patients with ATTR-CM treated with patisiran, mean population-level differences of 7 to 8 m in 6MWT have practical relevance. The magnitude of the impact of patisiran on 6MWT performance over 12 months in APOLLO-B was associated with preserving the ability to perform activities of daily living.

Transthyretin (TTR) amyloidosis (ATTR) is a rapidly progressive, debilitating, and fatal multisystem disease caused by misfolded TTR accumulating as toxic amyloid deposits in the nerves, heart, gastrointestinal tract, and musculoskeletal tissues.^1^, ^2^, ^3^ ATTR amyloidosis manifests with either cardiomyopathy (ATTR-CM), polyneuropathy, or both.^1^^,^^4^^,^^5^ ATTR amyloidosis is caused by inherited *TTR* gene variants that encode unstable protein in hereditary ATTR (ATTRv, v for variant) amyloidosis or is associated with aging in wild-type ATTR (ATTRwt) amyloidosis.^1^^,^^2^ Ongoing amyloid deposition in ATTRv and ATTRwt cardiomyopathy drives worsening heart failure (HF), arrhythmias, and conduction disease, as well as a steady decline in functional capacity and quality of life (QOL).^6^, ^7^, ^8^, ^9^ Outcomes in ATTR-CM have improved with earlier diagnosis and improved HF management in recent years, and novel therapies are expected to improve morbidity and mortality further.^10^^,^^11^ As survival increases, a greater emphasis on preserving functional capacity can be expected. Clinical studies in ATTR-CM commonly employ the 6-minute walk test (6MWT) as a measure of functional capacity; however, there is limited understanding of how changes in the 6MWT distance in clinical trials translate to the daily lives of patients with ATTR-CM. An estimate of the minimal clinically important difference (MCID) for the 6MWT in a contemporary population with ATTR-CM would help to relate treatment benefits measured in a clinical trial setting to patients' real-world experiences.

Patisiran is an RNA interference therapeutic designed to degrade variant and wild-type *TTR* messenger RNA in the hepatocyte, thereby resulting in rapid knockdown of circulating TTR protein. In the phase 3, randomized, double-blind, placebo-controlled APOLLO-B study in patients with ATTR-CM, the impact of patisiran on decline in functional capacity and health status was assessed with the 6MWT and the cardiomyopathy-specific Kansas City Cardiomyopathy Questionnaire-Overall Summary (KCCQ-OS), respectively.^12^ Patisiran demonstrated a statistically significant improvement in functional capacity compared with placebo, evidenced by a treatment benefit with patisiran in the 6MWT at month 12 (Hodges–Lehmann estimate of median difference: 14.69 [95% CI: 0.69-28.69] m).^12^ However, the magnitude of change in 6MWT distance that has a meaningful impact on functional capacity for patients with ATTR-CM is unknown.

The present analyses aim to relate the treatment benefits observed with patisiran in APOLLO-B to patients' daily lives. In support of this aim, we estimated the MCID for 6MWT in patients with ATTR-CM and developed a comparison framework with the KCCQ-OS and a series of activities of daily living (ADLs) that demand increasingly greater physical effort. These analyses can help to interpret the impact of changes in functional capacity as quantified by 6MWT in APOLLO-B on the daily lives of patients with ATTR-CM and the practical benefits of preserving functional capacity.

## Methods

### Study design

APOLLO-B was an international, phase 3, randomized, placebo-controlled, multicenter study that evaluated the efficacy and safety of patisiran in patients with ATTR-CM (ATTRv or ATTRwt) in a 12-month, double-blind period. Full details of the design and patient population for this study have been reported previously.^12^ The APOLLO-B trial conformed with the principles outlined in the Declaration of Helsinki.^13^

### Outcomes

We assessed the change in functional capacity, as measured by the change from baseline in distance covered in the 6MWT at month 12. Participants' health status and QOL were measured by KCCQ-OS, a well-validated, 23-item, disease-specific health status measure that integrates the impact of HF on a patient's physical limitations, total symptoms, social limitations, and QOL. Scores range from 0 to 100; scores of 0 to 24 indicate very poor to poor, 25 to 49 poor to fair, 50 to 74 fair to good, and 75 to 100 good to excellent health status.^14^ KCCQ-OS scores have been associated with survival, hospitalizations, and costs in HF populations.^15^^,^^16^ Multiple studies across diverse etiologies of HF have established that a change of 5 points in KCCQ-OS is clinically significant, whereas 10 to 15 points represents a moderate-to-large clinical change and >15 points a large-to-very-large change. In addition, the individual items of the KCCQ Physical Limitation scale quantify patients' ability to perform routine activities, ranging from low exertional requirements (eg, dressing oneself) to more demanding activities (eg, jogging or hurrying to catch a bus). Ability to perform ADLs is quantified on a 5-point Likert scale ranging from 1 = “extremely limited” to 5 = “not limited at all.”

### Statistical analyses

We calculated the MCID for 6MWT by anchoring the changes in 6MWT to established categories of clinically significant change defined by KCCQ-OS score. The median change from baseline in 6MWT distance at month 12 was calculated in categories of patients who showed a change from baseline to month 12 in KCCQ-OS score of ≤−20, >−20 to ≤−10, >−10 to ≤−5, >−5 to <+5, and ≥+5 to <+10, ≥+10 to <+20, and ≥+20 points, which correspond to a large, moderate, and small deterioration; stable; and small, moderate, and large improvement in HF status, respectively.^17^ The MCIDs for deterioration and improvement were estimated as the difference between the median 6MWT changes in the deterioration or improvement categories, respectively, and the stable KCCQ-OS category. The IQR for the MCID was calculated through a bootstrap of 10,000 samples. A sensitivity analysis using the KCCQ Physical Limitation score as an anchor was performed using the same methodology.

To characterize the relationship between change in 6MWT distance and performance in ADLs, we fit a proportional-odds cumulative logit model for each item in the KCCQ Physical Limitation domain. A change in magnitude of +15 m preservation in the 6MWT was selected as the basis for calculating relative odds of ADL worsening, as it was the treatment effect observed in the APOLLO-B trial. The model included the following covariates: baseline 6MWT as a continuous covariate, baseline KCCQ ADL score and study visit as a factor, and the change from baseline in 6MWT by study visit interaction. Missing data were assumed to be missing completely at random. Patients who selected the KCCQ response option indicating that they did not perform the ADL of interest or were limited in that ADL for reasons unrelated to the disease were treated as missing.

The study is being conducted in accordance with all applicable regulatory requirements, the current guidelines of Good Clinical Practice, and principles that have their origin in the Declaration of Helsinki. The institutional review board or independent ethics committee at each center approved the study protocol and amendments as previously described.^12^ All patients provided written informed consent.

## Results

### Patients

The APOLLO-B trial randomized 360 patients 1:1 to receive placebo (n = 179) or patisiran (n = 181). The patient demographics and baseline disease characteristics have been described in detail.^12^ In brief, the median age of patients was 76 years, and most of them were men. Around 80% of patients had ATTRwt. Demographics and clinical characteristics were well balanced between treatment groups.

### Clinical meaningfulness of changes in functional capacity

**Figure 1:**
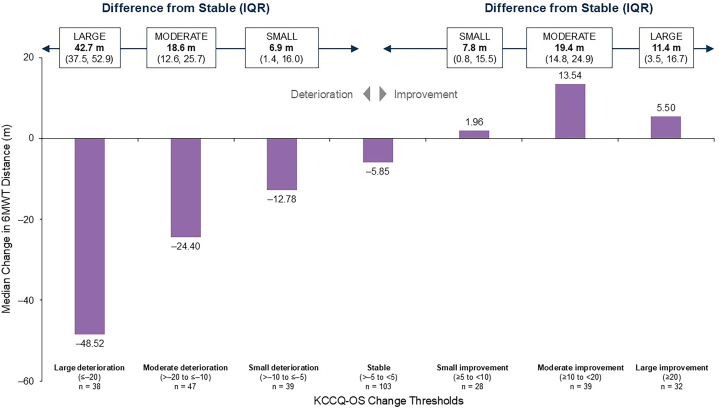
Change in 6-Minute Walk Test Distance by Change in Kansas City Cardiomyopathy Questionnaire-Overall Summary This graph shows the average change in 6MWT distance among patients with large, moderate, and small changes (improvements and deteriorations, respectively) in KCCQ-OS from baseline to month 12 in APOLLO-B. In addition, the graph presents 6MWT MCID values, estimated as the difference between a given KCCQ-OS change category and the ‘stable’ KCCQ-OS category with respect to average 6MWT change from baseline. Data are presented for the total population (ie, patisiran and placebo groups combined). 6MWT = 6-minute walk test; KCCQ-OS = Kansas City Cardiomyopathy Questionnaire-Overall Summary; MCID = minimal clinically important difference.

Patients who had large, moderate, and small deteriorations in KCCQ-OS had median changes in 6MWT distance of −48.52 m, −24.40 m, and −12.78 m, respectively, at month 12 (Figure 1). Patients who remained stable in KCCQ-OS had a median change in 6MWT distance of −5.85 m, and those who had small, moderate, and large improvements in KCCQ-OS had +1.96 m, +13.54 m, and +5.50 m changes in 6MWT distance, respectively. By comparing the change in 6MWT distance in patients having a large, moderate, or small deterioration in KCCQ-OS with those remaining stable, associated differences in worsening 6MWT distance of 42.7 (IQR: 37.5, 52.9) m, 18.6 (IQR: 12.6, 25.7) m, and 6.9 (IQR: 1.4, 16.0) m, respectively, were calculated. Additionally, comparison of 6MWT distance in patients having a small, moderate, or large improvement in KCCQ-OS with those remaining stable resulted in associated differences for an improved 6MWT of 7.8 (IQR: 0.8, 15.5) m, 19.4 (IQR: 14.8, 24.9) m, and 11.4 (IQR: 3.5, 16.7) m, respectively. Hence, the estimated MCID for a small but clinically significant deterioration or improvement are 6.9 and 7.8 m. The sensitivity analysis using the KCCQ Physical Limitation Score as an anchor estimated an MCID of 6.7 m and 10.4 m for patients who improved or worsened in the 6MWT, respectively.

### Association between functional capacity and KCCQ ADLs

The association of walking preservation with ADLs may be described with a hypothetical example comparing 1 patient with a given level of walking preservation (as measured by change from baseline in 6MWT at month 12) with another patient with better walking preservation by a margin of ≥15 m. Based on estimates from the cumulative logit model, better walking preservation by a margin of ≥15 m was associated with lower odds of worsening (ie, better preservation) in ADLs based on an ordinal scale: ability to walk 1 block (OR: 0.88 [95% CI: 0.83-0.93]), climb stairs (OR: 0.84 [95% CI: 0.80-0.89]), hurry or jog (OR: 0.88 [95% CI: 0.83-0.93]), dress oneself (OR: 0.85 [95% CI: 0.81-0.90]), and perform yard/housework or carry groceries (OR: 0.89 [95% CI: 0.84-0.93]) (Table 1).

**Table 1:** Association Between Change in 6MWT Distance and Performance on ADLs

Outcome of Worsening Ability to	OR (95% CI) per 15 m More Favorable Change in 6MWT Distance at Month 12^a^
1a Dress oneself	0.85 (0.81-0.90)
1b Shower/bathe	0.87 (0.82-0.93)
1c Walk 1 block on level ground	0.88 (0.83-0.93)
1d Perform yard/housework or carry groceries	0.89 (0.84-0.93)
1e Climb stairs without stopping	0.84 (0.80-0.89)
1f Hurry or jog	0.88 (0.83-0.93)

6MWT = 6-minute walk test; ADL = activity of daily living; KCCQ = Kansas City Cardiomyopathy Questionnaire.

a The model included the following covariates: baseline 6MWT as a continuous covariate, baseline KCCQ ADL score and study visit as a factor, and the change from baseline in 6MWT by study visit interaction.

### Treatment response in functional capacity, health status, and QOL

**Figure 2:**
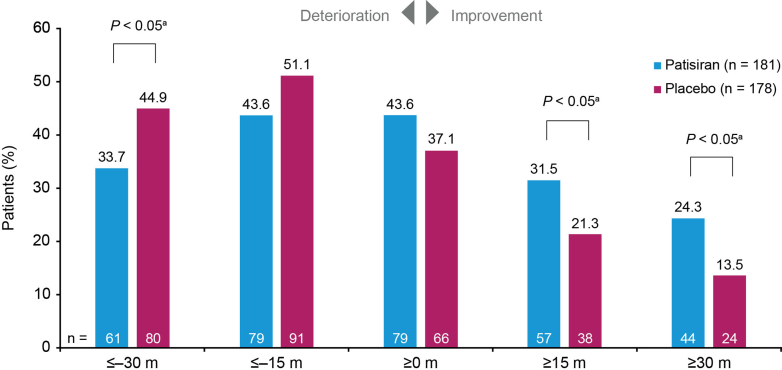
Patients With Specified Threshold Levels of Change in 6-Minute Walk Test This bar graph shows 6MWT changes in patients receiving patisiran and placebo from baseline to month 12 in APOLLO-B, with *P* values for significance of difference between patisiran and placebo. Results were obtained from an analysis of observed and imputed 6MWT data; for each patient, the change from baseline is averaged across 100 complete datasets. ^a^*P* values were calculated using the Cochran–Mantel–Haenszel test stratified by baseline tafamidis use. 6MWT = 6-minute walk test.

A greater proportion of patisiran-treated patients achieved specified improvements in 6MWT distance across a range of thresholds that exceeded the estimated MCID, compared with placebo-treated patients at month 12 (Figure 2). Significantly more patisiran-treated patients than placebo-treated improved by ≥15 m (32% vs 21%, *P* < 0.05) or ≥30 m (24% vs 14%, *P* < 0.01). Conversely, a greater proportion of placebo-treated patients had worsening of 6MWT distance compared with patisiran-treated patients at month 12 (Figure 2); significantly more placebo-treated patients than patisiran-treated worsened by >30 m (45% vs 34%, *P* < 0.05).

**Figure 3:**
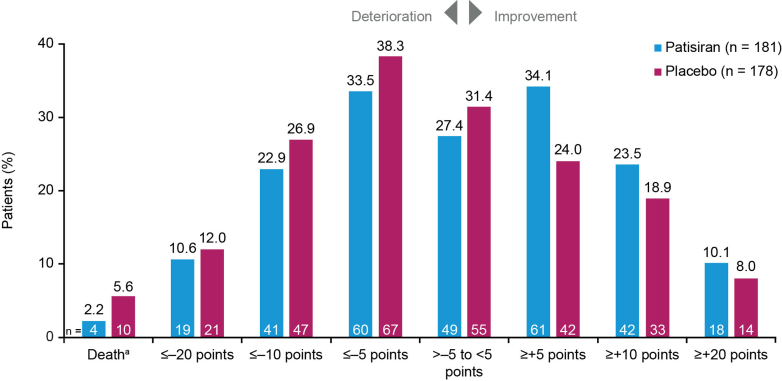
Patients With Specified Threshold Levels of Change in Kansas City Cardiomyopathy Questionnaire-Overall Summary The bar graph summarizes the percentage of patients experiencing different threshold levels of KCCQ-OS change from baseline to month 12 among those receiving patisiran and placebo in APOLLO-B. ^a^Deaths include heart transplants—placebo n = 2 (1.1%); patisiran n = 0 (0%)—and exclude one COVID death in the patisiran arm. KCCQ-OS = Kansas City Cardiomyopathy Questionnaire-Overall Summary.

Similarly, a greater proportion of patisiran-treated patients achieved specified improvements across a range of clinically meaningful thresholds in KCCQ-OS score at month 12 compared with placebo (Figure 3). Significantly more patisiran-treated patients than placebo-treated improved by ≥+5 points (34% vs 24%, *P* < 0.05). A greater proportion of placebo-treated patients showed a deterioration by 5 or more, 10 or more, and 20 or more points compared with patisiran, and a greater proportion of placebo-treated patients than patisiran-treated died by month 12 (Figure 3).

**Figure 4:**
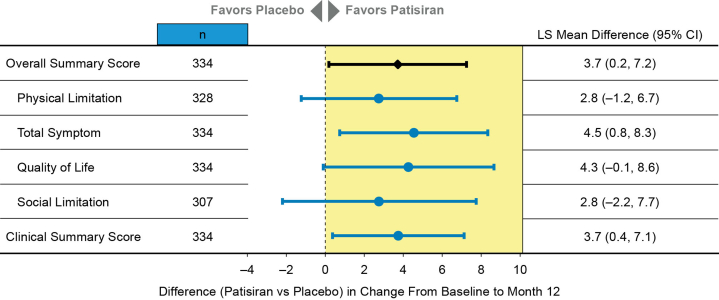
Treatment Differences in Change From Baseline in Kansas City Cardiomyopathy Questionnaire Scores This forest plot summarizes the LS mean differences between patients receiving patisiran and placebo with respect to KCCQ-OS score change and individual KCCQ domain score or subscore changes from baseline to month 12 in APOLLO-B. KCCQ = Kansas City Cardiomyopathy Questionnaire; KCCQ-OS = KCCQ-Overall Summary; LS = least squares.

**Figure 5:**
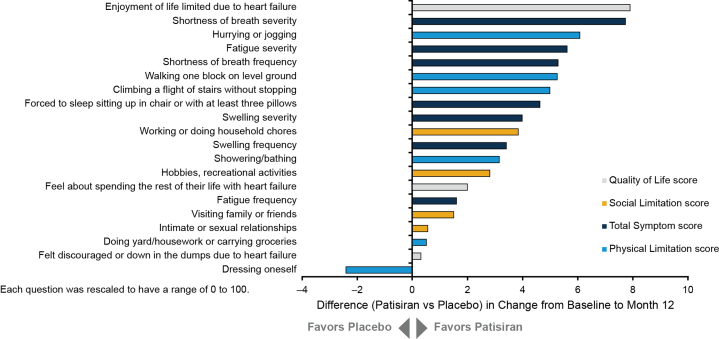
Mean Treatment Difference in Change From Baseline in Kansas City Cardiomyopathy Questionnaire-Overall Summary Questions This chart summarizes the differences between patients receiving patisiran and placebo with respect to changes in individual KCCQ-OS question scores from baseline to month 12 in APOLLO-B. KCCQ-OS = Kansas City Cardiomyopathy Questionnaire-Overall Summary.

Patisiran demonstrated a statistically significant benefit in health status and QOL (KCCQ-OS) compared with placebo at month 12 (least squares mean difference: 3.7 [95% CI: 0.2-7.2]; Figure 4). The treatment benefit with patisiran was also observed across all 4 domains of the KCCQ and the KCCQ Clinical Summary score. Responses to individual KCCQ-OS questions consistently favored treatment with patisiran over placebo, except for the ability to dress oneself (Figure 5). Among the largest treatment effects observed were those related to walking and demanding physical activities, such as hurrying or jogging and climbing stairs, as well as those related to symptoms that limit exertion, such as shortness of breath and fatigue. The greatest treatment effect was observed in the question related to the impact of HF on the patient's enjoyment of life.

## Discussion

Here, we aimed to interpret changes in 6MWT performance over 12 months among patients with ATTR-CM in the context of health status and QOL as well as ADLs. The goal was to obtain a practical understanding of the effect of patisiran on functional capacity from the APOLLO-B study. We estimated the MCID for 6MWT distance associated with a meaningful change in QOL in this contemporary population to be ∼7 m to 8 m, based on a KCCQ-OS-anchored calculation. Preservation of functional capacity, as measured by the 6MWT, was associated with preservation of the ability to perform ADLs. Overall, these analyses support the findings that treatment benefit with patisiran of +15 m in 6MWT distance compared with placebo in the APOLLO-B study translates to benefits in the daily lives of patients.^12^ Furthermore, patients treated with patisiran are more likely to have clinically meaningful improvements in health status and QOL than those treated with placebo, based on established thresholds for small, moderate, and large changes in KCCQ-OS score.

The 6MWT has previously been shown to correlate with KCCQ Physical Limitation and Clinical Summary scores in patients with chronic HF.^18^ Here, we demonstrate that better preservation of 6MWT distance is associated with greater preservation in the ability to perform basic ADLs and higher-order physical activities, including the ability to walk 1 block, climb stairs, hurry or jog, dress oneself, and perform yard/housework or carry groceries. Preserving function in 6MWT via therapeutic intervention may thus be associated with conserving the ability to perform everyday activities in this patient population. This, in turn, may aid in the retention of independent living, as loss of ability to conduct ADLs is correlated with increased risk of institutionalization.^19^

The current analysis also demonstrates that patients treated with patisiran have increased odds of clinically meaningful improvement in functional capacity as measured by 6MWT at 12 months compared with placebo; patisiran-treated patients have ∼70% greater odds of having improvement of ≥15 m in the 6MWT and over twice the odds of improving by ≥30 m. Therefore, patisiran-treated patients have greater odds of improved functional capacity, and this is likely to be associated with significant benefits for ADL performance. For example, the +15 m mean treatment benefit from baseline to 12 months with patisiran over placebo in APOLLO-B translates to 15.7% lower odds of worsening performance climbing stairs without stopping. Interestingly, underscoring the relevance of the treatment benefit of patisiran on 6MWT, the magnitude of this benefit is consistent with preventing the additional 10.7 m/year decline in 6MWT distance that is observed following an outpatient worsening HF event, as defined by an intensification of oral loop diuretic therapy, which is associated with all-cause mortality and recurrent cardiovascular events in patients with ATTR-CM.^20^^,^^21^ The clinical relevance of these treatment effects is further underscored by the more favorable outcomes with patisiran vs placebo in various KCCQ threshold analyses, as well as domain-level and item-level KCCQ analyses.

To our knowledge, this is the first report of a 6MWT MCID in an ATTR-CM population. The results help to translate the treatment effects on functional capacity measured in a clinical trial to benefits in ADL. The MCIDs for 6MWT vary widely between diseases because the MCID depends on the basis of disease, baseline 6MWT performance, and patients' demographic and disease characteristics.^22^^,^^23^ Thus, previously reported 6MWT MCIDs in other conditions do not necessarily apply to ATTR-CM, while the present results are specific to the typically older patient with ATTR-CM. The needs and expectations of such patients to preserve functional capacity and QOL will only become more important as cardiovascular outcomes and survival improve. In this regard, the magnitude of 6MWT decline that is prognostic for mortality and that is substantially greater than the MCID reported here is a less sensitive threshold for monitoring the contemporary population of patients with ATTR-CM.^21^

### Study limitations

These analyses were conducted post hoc and are considered hypothesis generating. In addition, our findings regarding the association of 6MWT distance with ADL performance were determined using data from the APOLLO-B population of patients with ATTR-CM, who were diagnosed and treated at earlier stages of the disease than patients in the past.^10^ APOLLO-B did not include a direct assessment of patients' abilities to perform ADLs; consequently, our analyses were limited to patient-reported responses to questions from the Physical Limitations domain of the KCCQ that assess functional capacity in a daily context. The estimated 6MWT MCID was based on the KCCQ-OS, a patient-reported assessment of the impact of HF on health status and QOL. Other approaches could yield different estimates. Although the cutoffs in 6MWT and KCCQ-OS that were used as analytical reference points or anchors were based on the treatment difference with patisiran^12^ and established standard categories of deterioration,^17^ respectively, these measures are both continuous and any cutoffs could be considered arbitrary. Furthermore, APOLLO-B inclusion criteria required patients to walk ≥150 m during the 6MWT at screening. The estimated 6MWT MCID may not apply to patients who would have been excluded for this reason. It is conceivable, however, that seemingly modest, absolute declines are associated with even greater impacts on ADLs and QOL among patients who are more severely limited. Finally, we note that patisiran is not approved for the treatment of ATTR-CM in the United States but is approved in Brazil and France, where, in the latter case, patisiran is approved for compassionate use in patients failing tafamidis 61 mg.

## Conclusions

In this analysis in patients with ATTR-CM, the 6MWT MCID was ∼7 m to 8 m, suggesting that this amount of change in 6MWT distance is clinically meaningful to patients and is associated with negative or positive impacts on their health status and QOL. Therefore, the 15 m treatment difference observed with patisiran at 12 months in APOLLO-B suggests a meaningful benefit on functional capacity. It is also positively associated with preserved ability to perform everyday activities and is accompanied by other QOL benefits, such as protection against the negative impacts of HF on the enjoyment of life. Overall, the benefit observed with the 6MWT in APOLLO-B translates into a clinically meaningful impact in the lives of patients (Central Illustration).

Perspectives **COMPETENCY IN MEDICAL KNOWLEDGE:** As survival and cardiovascular outcomes in ATTR-CM improve with earlier diagnosis and the emergence of novel therapies, a greater emphasis on preserving functional capacity can be expected. There is poor understanding of how improvement in functional capacity in clinical trials translates to the daily lives of patients.

**TRANSLATIONAL OUTLOOK:** The meaningful treatment benefit of patisiran in APOLLO-B on the 6MWT distance translated into preserved ability to perform everyday activities and was accompanied by other QOL benefits. These findings suggest that knockdown of circulating TTR protein by RNAi therapeutics may have a positive impact on patients' enjoyment of life and future independence.

**Central Illustration:**
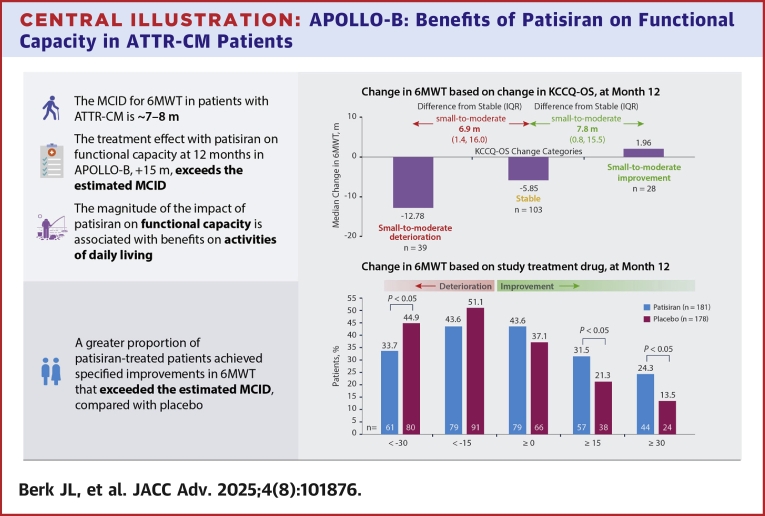
APOLLO-B: Benefits of Patisiran on Functional Capacity in ATTR-CM Patients Functional capacity in the phase 3 randomized, placebo-controlled, 12-month APOLLO-B study was measured by the 6MWT, and health status and QOL by the KCCQ-OS. The MCID for 6MWT was estimated as the difference between the median change in KCCQ-OS categories of small-to-moderate deterioration, stable, and improvement. 6MWT = 6-minute walk test; ATTR-CM = transthyretin amyloidosis with cardiomyopathy; KCCQ-OS = Kansas City Cardiomyopathy Questionnaire-Overall Summary; MCID = minimal clinically important difference; QOL = quality of life.

## Funding support and author disclosures

This work was supported by 10.13039/100006400Alnylam Pharmaceuticals. Dr Berk has received research funding from 10.13039/100006400Alnylam Pharmaceuticals, BridgeBio/Eidos Therapeutics, and 10.13039/100013669Ionis Pharmaceuticals; consulting fees from Alexion Pharmaceuticals, BridgeBio/Eidos Therapeutics, Intellia Therapeutics, Ionis Pharmaceuticals/AstraZeneca, and Purpose Pharma; payment or honoraria for lectures, presentations, Speakers Bureaus, or educational events from MERCKI, NeurologyLive, and UpToDate; advisory board membership for Alexion Pharmaceuticals, BridgeBio/Eidos Therapeutics, Intellia Therapeutics, and Ionis Pharmaceuticals/AstraZeneca; and unpaid leadership or fiduciary role in other board, society, committee, or advocacy group in the Amyloid Support Group and International Society of Amyloidosis (ISA). Dr Lairez has received consulting fees from Alnylam Pharmaceuticals, AstraZeneca, and Pfizer. Dr Schwartzmann has received consulting or personal lecture fees from Alnylam Pharmaceuticals, AstraZeneca, Bayer, Boehringer Ingelheim, Novartis, and Pfizer. Drs Bender, White, and Jay, and Mr Danese are employed by Alnylam Pharmaceuticals and report ownership of Alnylam Pharmaceuticals shares. Dr Witteles has received research funding from 10.13039/100006400Alnylam Pharmaceuticals, BridgeBio, Ionis Pharmaceuticals, Intellia Therapeutics, and Janssen; and consulting fees from Alexion, Alnylam Pharmaceuticals, AstraZeneca, BridgeBio, Novo Nordisk, and Pfizer.
